# Fabrication and Evaluation of Porous Beta-Tricalcium Phosphate/Hydroxyapatite (60/40) Composite as a Bone Graft Extender Using Rat Calvarial Bone Defect Model

**DOI:** 10.1155/2013/481789

**Published:** 2013-12-17

**Authors:** Jae Hyup Lee, Mi Young Ryu, Hae-Ri Baek, Kyung Mee Lee, Jun-Hyuk Seo, Hyun-Kyung Lee

**Affiliations:** ^1^Department of Orthopedic Surgery, College of Medicine, SMG-SNU Boramae Medical Center, Seoul National University, Seoul 156-707, Republic of Korea; ^2^Institute of Medical and Biological Engineering, Seoul National University Medical Research Center, Seoul 110-799, Republic of Korea; ^3^Research Center, Bioalpha, Sung-Nam 462-120, Republic of Korea

## Abstract

Beta-tricalcium phosphate (**β**-TCP) and hydroxyapatite (HA) are widely used as bone graft extenders due to their osteoconductivity and high bioactivity. This study aims to evaluate the possibility of using porous substrate with composite ceramics (**β**-TCP: HA = 60% : 40%, 60TCP40HA) as a bone graft extender and comparing it with Bio-Oss. Interconnectivity and macroporosity of **β**-TCP porous substrate were 99.9% and 83%, respectively, and the macro-porosity of packed granule after crushing was 69%. Calvarial defect model with 8 mm diameter was generated with male Sprague-Dawley rats and 60TCP40HA was implanted. Bio-Oss was implanted for a control group and micro-CT and histology were performed at 4 and 8 weeks after implantation. The 60TCP40HA group showed better new bone formation than the Bio-Oss group and the bone formation at central area of bone defect was increased at 8 weeks in micro-CT and histology. The percent bone volume and trabecular number of the 60TCP40HA group were significantly higher than those of Bio-Oss group. This study confirms the usefulness of the porous 60TCP40HA composite as a bone graft extender by showing increased new bone formation in the calvarial defect model and improved bone formation both quantitatively and qualitatively when compared to Bio-Oss.

## 1. Introduction

The necessity of bone graft substitute is gradually increasing in the field of maxillofacial surgery and orthopaedic surgery to improve bone defect healing and bone fusion. The majority of the bone graft substitute are bone graft extenders for reducing autologous bone usage since they have similar effect as autologous bone. Bone morphogenetic proteins are osteoinductive bone substitutes which can replace autologous bone [1]. Osteoconductive materials are mainly used as bone graft extenders and hydroxyapatite (HA) is a representative osteoconductive material with similar composition as bone. HA has been frequently used as a bone graft in spine fusion since it is biocompatible and can make chemical bondings with surrounding bones [2, 3]. However, it is brittle and hard to achieve complete remodeling due to low level of resorption after insertion. Thus, bone graft extenders with high resorption level have been developed and some of the products are made of *β*-tricalcium phosphate (*β*-TCP), calcium pyrophosphate, and bioactive glass-ceramics [4–7]. Amongst them, *β*-TCP has long been used as a bone graft extender and is known to have high osteoconductivity [8].

While the material with high resorption has low risk as a foreign material since it does not stay inside of body, but bone fusion or bone healing rate can be lowered if the resorption occurs before new bone formation. To complement the limitation, the composite materials of *β*-TCP with high resorption and HA with good osteoconductivity have been examined in vitro and in vivo and shown the results about their usefulness [9–11]. Bio-Oss (Geistlich Pharma North America, Inc., Princeton, USA) is a bone substitute made of bovine bone and mainly used for dental bone regeneration, dental implant therapy, and periodontal defect. It is known to be effective in maxillofacial surgery for bone regeneration [12] and capable of volume preservation [13], and has good long-term results [13–15].

Thus, this study aims to evaluate the usefulness of the composite material, 60TCP40HA (*β*-TCP : HA = 60% : 40%), as a bone graft extender in rat calvarial bone defect model by comparing it with Bio-Oss.

## 2. Materials and Methods

### 2.1. Preparation of Porous Ceramic Granules

Bone graft substitutes with calcium phosphates such as HA, *β*-TCP, and biphasic bone substitute were produced with pure, medical device-class HA (Cerectron Co., Kimpo, South Korea) and *β*-TCP (RN2 Technology, Pyeongtaek, South Korea). Raw materials of HA and *β*-TCP were provided as specified in ASTM F1185-03 [16] and ASTM F1088-04 [17], respectively, and followed the standard specification for surgical implants.

*β*-TCP 60 wt% and HA 40 wt% were mixed with zirconia balls and ethanol and then subjected to ball mill to form a homogenous composite. After drying up ethanol, the composite was sintered at every 50°C within a range of 1100~1300°C to determine the sintered temperature. Relative density, crystal shape, and microstructure of the sintered body were considered for the sintered temperature. Once the temperature set up, the dried powder was homogenously mixed with specific vehicle using 3-roll mill and permeated into polyurethane sponge with 60 ppi (pore per inch) of porosity to shape porous substrate. The porous substrate was dried at 120°C for 24 hours and sintered at the set-up temperature for 2 hours. The sintered substrate was crushed to a size of 0.6~1.0 mm with a ceramic knife and allocated. Finally, the substrate was packed to keep the porous structure and sterilized with 25~40 KGy of gamma radiation.

#### 2.1.1. Mechanical Properties Analysis

The relative density of the sintered body was calculated using Archimedes's principle and compared with theoretic density of *β*-TCP, 3.14 g/cm^3^. Crystal shape of the sintered body was attained through X-ray diffraction (D8FOCUS (2.2 kW), Bruker AXS, Berlin, Germany) and microstructure was evaluated with field emission scanning electron microscope (FE-SEM, JSM-6700F, Jeol, Tokyo, Japan). Content of HA in the 60TCP40HA composite was quantitated by the calculation method suggested in ISO 13779-03 [18].

Porosity of the porous bone graft was evaluated by micro-CT (SKYSCAN 1173, SKYSCAN, Kontich, Belgium). To determine the porosity of 60TCP40HA after crushing, melting paraffin was packed with the crushed granule and subjected to micro-CT to achieve three-dimensional (3D) image. The cube with 3 mm of each side was set inside of the 3D image and macroporosity was calculated based on the bone volume estimated within a range of the cube.

### 2.2. In Vivo Study

#### 2.2.1. Animals and Implantation

Fifty-two male Sprague-Dawley rats were randomized into following two groups: Group I of 60TCP40HA and Group II of Bio-Oss. Each group was further divided into a 4-week group and an 8-week group and 13 rats were assigned to each small group. The animals were anesthetized with zoletil (0.4 mL/kg, Virbac Laboratories, Carros, France) and rumpun (10 mg/kg, Bayer Korea Ltd., Korea) and the region around scalp was shaved and antisepticized with betadine. Calvarial skin was incised longitudinally and periosteum was separated. To prevent spontaneous bone healing, an 8 mm trephine burr was used to generate calvarial defect followed by saline irrigation. The same amount (25 mg) of samples was implanted into the calvarial defect, periosteum and scalp were sutured. Cefazoline (100 mg) was given to the animals by intramuscular injection immediately after the surgery for 2 days. The animals were raised at 22 ± 5°C temperature and 50 ± 5% humidity without interruption and sacrificed at 4 weeks or 8 weeks after implant for analysis. All information regarding animal experiments was approved by the International Animal Care and Use Committeeon Ethics at the Clinical Research Institute of Seoul National University Hospital.

#### 2.2.2. Animals Micro-CT Evaluation

After sacrifice, overall region of cranium including the implant site was analyzed through micro-CT (Skyscan 1173, Belgium) under the condition of aluminum filter with 130 kV, 30 *μ*A, and 12.14 *μ*m. Thirty CT pictures per each group were evaluated with 7.99 mm, circular ROI. The taken images were reconstructed with axial, sagittal, and coronal plane. For the regions with bone loss but excluding implant site, percent bone volume (bone volume/total volume, BV/TV), bone surface/volume ratio (BS/BV), trabecular thickness (Tb.Th), trabecular separation (Tb.Sp), trabecular number (Tb.N), trabecular bone pattern factor (Tb.Pf), structure model index (SMI), and degree of anisotropy (DA) were measured and the values from both groups were compared.

#### 2.2.3. Histological Evaluation

The harvested specimens were dehydrated with ethanol sequentially and the dehydrated tissue sections were embedded in methyl methacrylate resin followed by solidification at 37.5°C for 4 weeks. Sections were generated in sagittal plane with 4 *μ*m thickness and covered the region including both normal calvaria and the calvarial defect with the implant. New bone formation, bone remodeling level, and absorption level of carrier in the region around the implant were evaluated through hematoxylin and eosin (H&E) staining and light microscopy.

### 2.3. Statistical Analysis

Two groups were compared using the *t*-test. *P* values <0.05 were considered statistically significant.

## 3. Results

In this study, the data was generated from a total of 49 rats since three rats died during this experiment. One rat from the 4-week group and two from the 8-week group. The cause of death was postoperative bleeding and wound infection.

### 3.1. (1) Phase Analysis and Porosity

**Figure 1:**
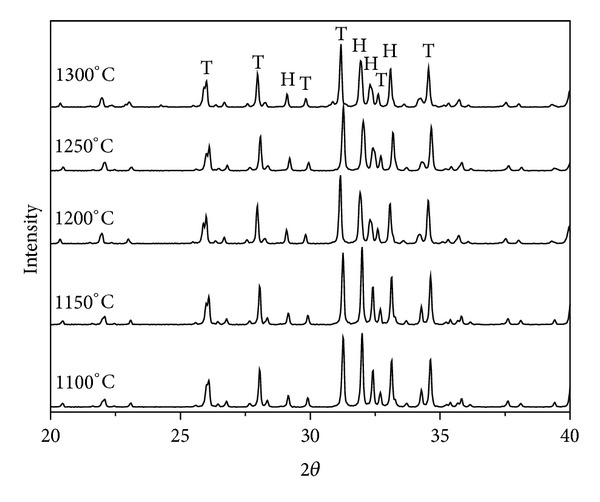
X-ray diffraction patterns of 60TCP40HA according to sintering temperature between 1100°C and 1300°C. T: tricalcium phosphate, H: hydroxyapatite, and 2*θ*: the take-off angle of the diffracted X-ray beam (spot) relative to the main beam.

**Figure 2:**
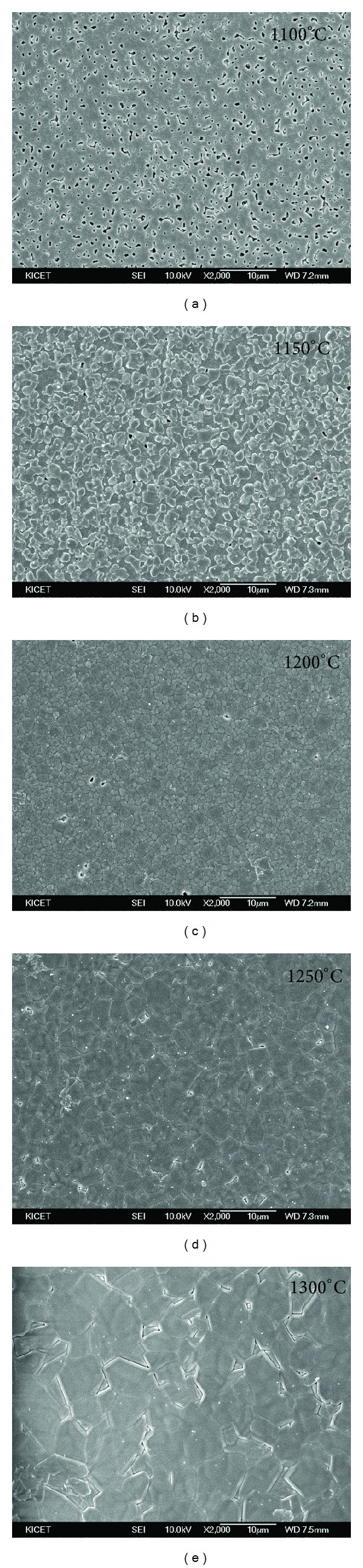
Micro-structure images of 60TCP40HA sintered body according to the temperature.

**Figure 3:**
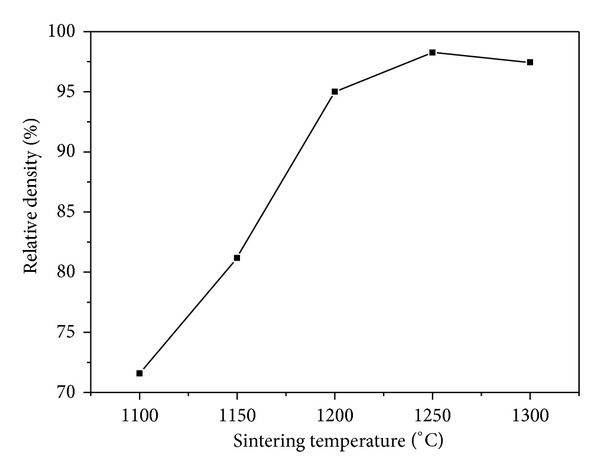
Relative density profile of 60TCP40HA sintered body according to sintering temperature.

The composite ceramic (60TCP40HA) was sintered at 1100~1300°C and the resulting powder was analyzed through X-ray diffraction (Figure 1). Other calcium phosphate compounds besides HA and *β*-TCP were not found at all temperature and CaO phase was not shown either. The diffraction intensity of HA and *β*-TCP were similar when sintered at 1100°C, but the intensity of HA was gradually decreased as the sintering temperature went up. The content of HA was calculated based on “qualitative and quantitative determination of the foreign phases” by international standard of medical devices, ISO 13779-03 (Table 1). The HA content was 57.6% at 1100°C, gradually decreased as the temperature went up and increased again at over 1200°C. In microstructure of the sample sintered at 1100°C, the pores were not removed, indicating that the sintering was not processed. From 1150°C, the sintering started to be processed and the pores were decreased (Figure 2). Up to 1250°C, the pores were gradually decreased and the sintering was well-processed due to grain growth. But at 1300°C, cracks occurred because of interior thermal shock. The relative density, which represents the density of the sintered body compared with theoretical density of *β*-TCP, was dramatically increased as the temperature rose and highest (98.3%) at 1250°C (Figure 3). Considering the HA content and the density of microstructure and sintering body, 1250°C was the optimal temperature for sintering of the 60TCP40HA composite bone substitute.

**Figure 4:**
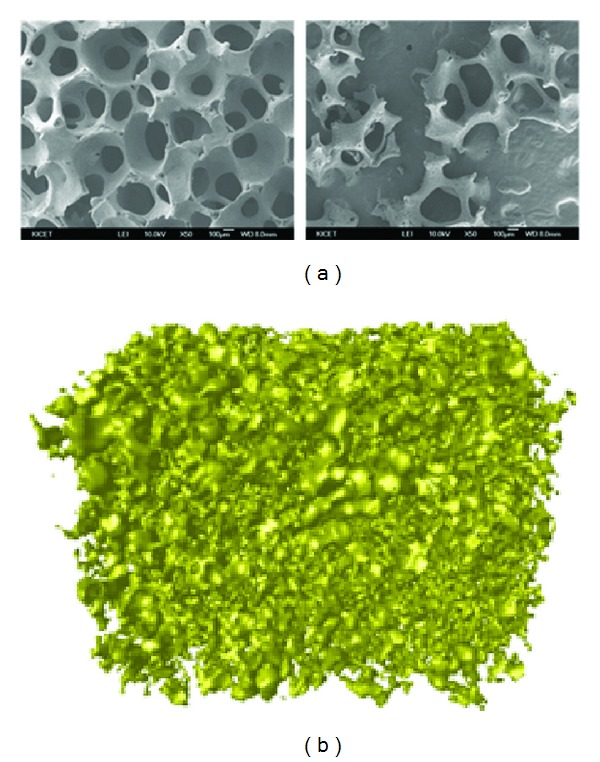
Micro-structure of 60TCP40HA. (a) SEM images of porous (left) substrate and (right) granules manufactured by sponge method. (b) Micro-CT image of packed granules which are in the range of 0.6 mm and 1.0 mm.

The porous substrate generated with 60 ppi sponge consisted of the connected pores with at least 300 *μ*m of diameter (Figure 4(a)). The porosity and pore interconnectivity of the substrate measured by micro-CT were 84% and 99.9%, respectively, inferring that the porous interior of the graft can provide spaces for bone growth. The granules crushed with a size of 0.6~1.0 mm also contained the internal pores, but the porosity confirmed by packing was decreased (Figure 4(b)). Indeed, porosity of the granule packed with melting paraffin was 68.8%, which represented about 15% reduced level.

### 3.2. (2) Micro-CT Results

**Figure 5:**
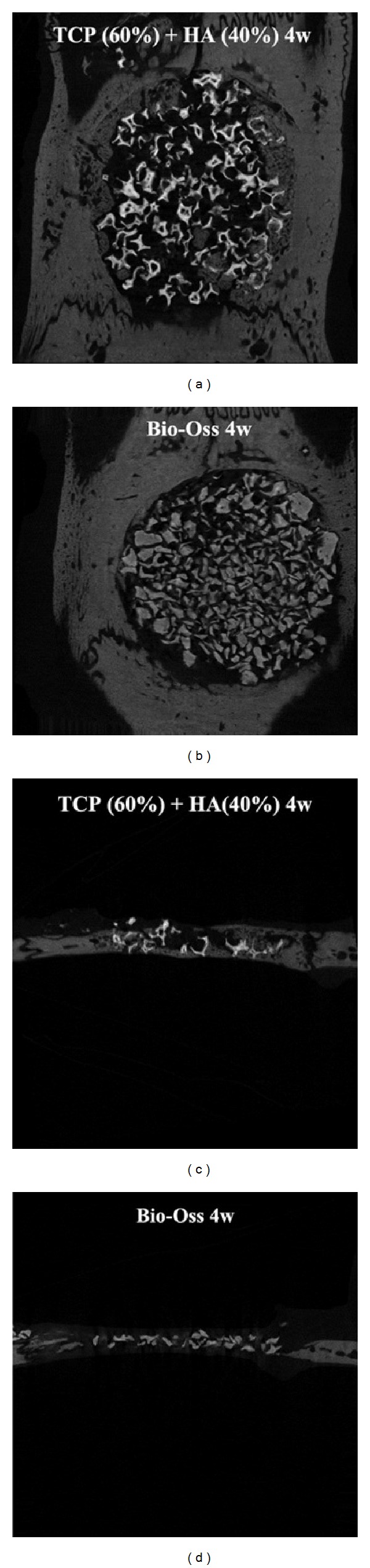
Micro-CT results of 60TCP40HA and Bio-Oss 4 weeks after implantation.

In the 4-week groups, both groups showed new bone formation in peripheral area of the calvarial defect. The Bio-Oss group showed Bio-Oss granules in central part of the defect but not bony tissues. However, the 60TCP40HA group formed new bone at central region of the defect. The specimens inserted in both groups kept the original shape and no degradation was found (Figure 5).

**Figure 6:**
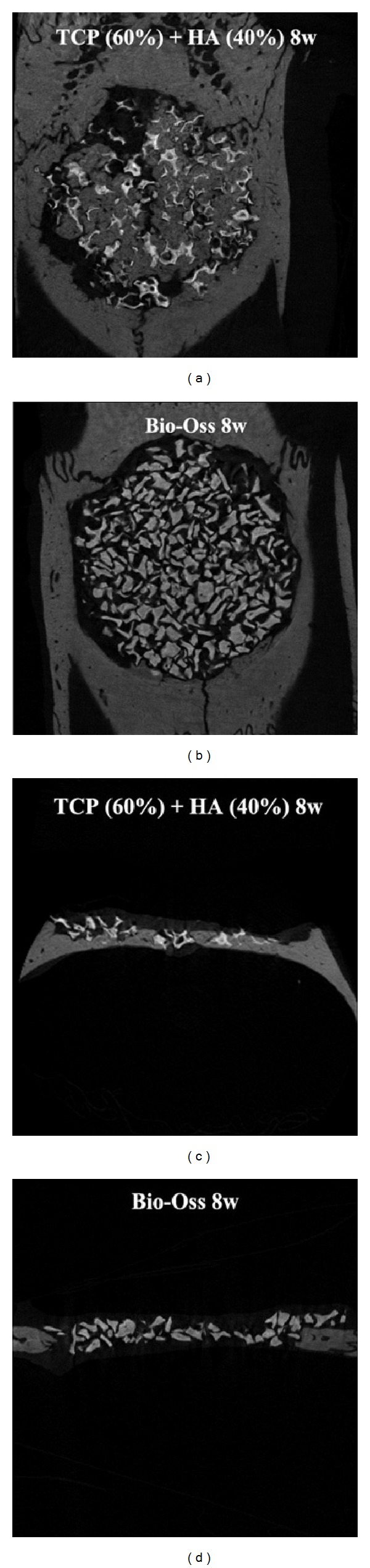
Micro-CT results of 60TCP40HA and Bio-Oss 8 weeks after implantation.

In the 8-week groups, bone formation in central region of the defect was increased in both groups compared to the 4-week groups. While the bone formation was marginal in central region of the defect from the Bio-Oss group, it was much clearer in the 60TCP40HA group, and quality of the new bone in sagittal image was similar to the one of surrounding bone. In the Bio-Oss group, boundary of the peripheral region became unclear and degradation was progressed. In the 60TCP40HA group, radio-opacity of implant sample was reduced and thickness of the pores became thinner, indicating more active degradation (Figure 6).

Among the parameters in micro-CT, percent bone volume was not significantly different between the 60TCP40HA group and the Bio-Oss group at 4 weeks. However, the volume was about twice in the 60TCP40HA group with statistical significance (*P* = 0.02) at 8 weeks. When compared with 4 weeks, the rate of increase at 8 weeks was also higher in the 60TCP40HA group (143.0%) compared to the level in the Bio-Oss group (40.4%) (Tables 2 and 3). The Bio-Oss group and the 60TCP40HA group showed higher trabecular number at 4 weeks and 8 weeks, respectively, but there were no statistical significance. The trabecular thickness was significantly higher in the 60TCP40HA group at 4 weeks (*P* < 0.00001) and also at 8 weeks (*P* = 0.0005).

### 3.3. (3) Histological Results

**Figure 7:**
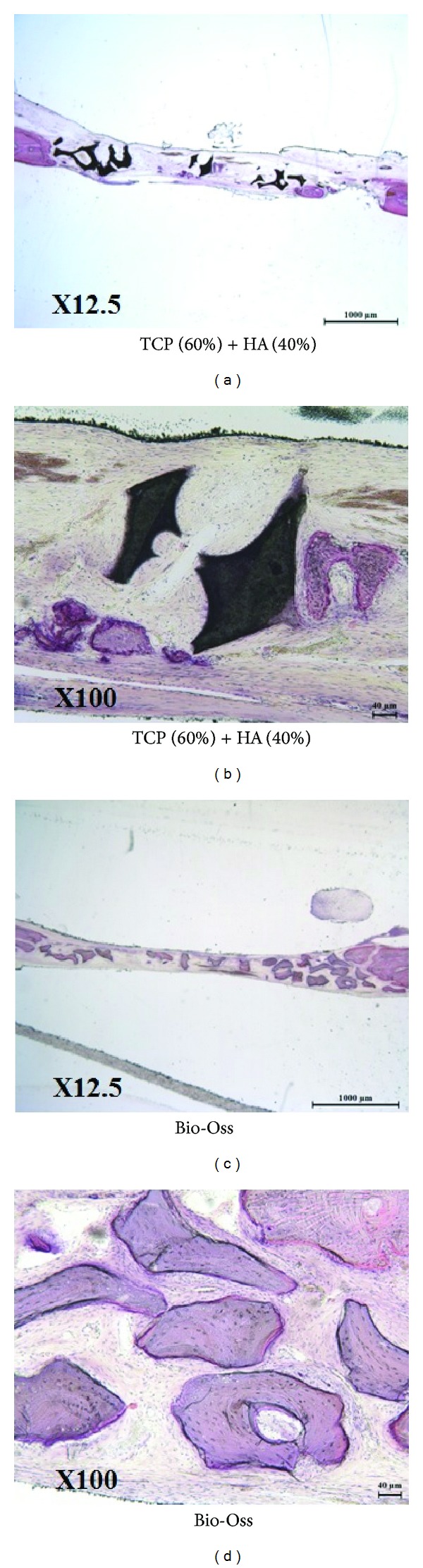
Undecalcified histology of 60TCP40HA and Bio-Oss 4 weeks after implantation.

In the 4-week groups, undecalcified histology found almost none of the bony tissue from both groups. Mixed form of bony tissues, cartilaginous tissues, and fibrous tissues was observed at around the specimen, but no bony tissues were shown at the center of calvarial defect (Figure 7).

**Figure 8:**
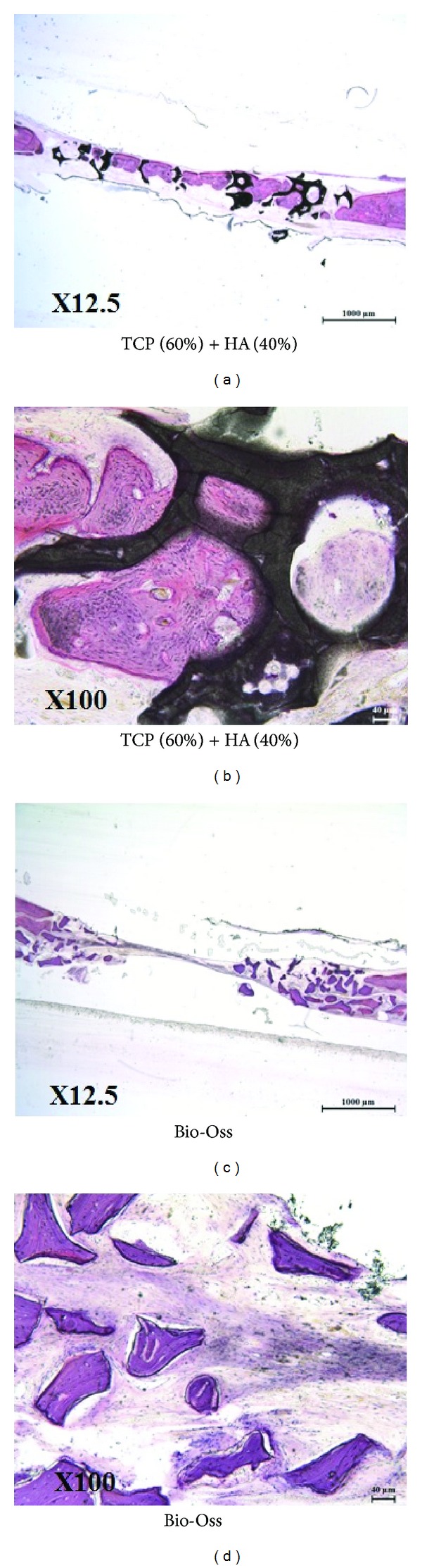
Undecalcified histology of 60TCP40HA and Bio-Oss 8 weeks after implantation.

In the 8-week groups, bony tissues were shown at central region of the defect in the 60TCP40HA group but only fibrous tissues were found in the Bio-Oss group. The bony tissues in the 60TCP40HA group connected between the samples and were observed inside of the pores. Also, quality of the newly formed bone was similar to the surrounding bone, confirming that the remodeling was processed (Figure 8).

## 4. Discussion

Bone graft extenders with porous ceramics have been used not only in the surgeries for bone defect or spine fusion after bone tumor operation but also in dental surgery and maxillofacial operation. The bone graft extenders with porous ceramics for small bone defect should be designed differentially in size or porous structure from the ceramics for spinal fusion operation. As the ceramics have higher porosity, they provide better environment for the growth of mesenchymal stem cells and perivascular tissues, but at the same time become more brittle. The weakness of this study is that we compared two materials with different architecture, therefore the differences obtained could be influenced by the different porosity of the materials. Micro-CT found no clear differences in peripheral area of calvarial defect between two groups, but the 60TCP40HA showed significantly higher volume of newly formed bone in central area. The difference was shown only at 8 weeks but not at 4 weeks, indicating that both groups had no clear difference initially, but as time passed, the 60TCP40HA group showed better bone formation possibly due to their porous structure and high osteoconductivity. 60TCP40HA is considered to be superior in incorporation between graft and host bone because it has 68.8% of porosity as a crushed granule form with a size of 0.6~1.0 mm as opposed to Bio-Oss which does not have porous structure. Higher percent bone volume and trabecular thickness of new bone in the 60TCP40HA group are considered that both quantity and quality of newly formed bone are improved in the group. In histology, new bone formation was marginal in both groups at 4 weeks, but it was significantly higher in the 60TCP40HA group at 8 weeks. These are consistent with the micro-CT results showing that both quantity and quality of new bone formation are significantly higher in the 60TCP40HA group. The better efficacy of the composite is possibly because tricalcium phosphate has higher osteoconductivity than bovine bone. Indeed, *β*-TCP is known to show osteoconductivity by *α*2*β*1 integrin and down-stream mitogen-activated protein kinase (MAPK)/extracellular related kinase (ERK) signaling pathway [19]. HA has been reported to have some level of osteoinductivity as well as osteoconductivity [20–22]. Since the TCP-HA composite is also reported to have osteoinductivity [23, 24], better new bone formation in the 60TCP40HA group can be due to superior osteoconductivity and osteoinductivity of the composite compared to Bio-Oss made of bovine bones.

The graft resorption was observed in both groups at 8 weeks. It is possibly because both grafts have small size (around 1 mm), and thus their surface area is relatively high. Compared to HA only, the composite contain 60% of *β*-TCP and it is also thought to contribute to the increased bone resorption.

## 5. Conclusion

In conclusion, the porous granules with 0.6~1.0 mm of the *β*-TCP 60%, HA 40% composite sintered at 1250°C were generated to have 68.8% of porosity with over 300 *μ*m pores. Compared to Bio-Oss confirmed to be effective in maxillofacial surgery, the composite showed improved new bone formation quantitatively and qualitatively in the rat calvarial defect model, suggesting its application as a bone graft extender.

## Figures and Tables

**Table 1:** HA contents of 60TCP40HA sintered body, which is calculated by ISO 13779-03.

Sintering temp.	1100°C	1150°C	1200°C	1250°C	1300°C

HA (%)	57.6	44.0	33.5	41.2	50.0

**Table 2:** Micro-CT results after 4 weeks of implantation.

Group (*n* = 7)	Average (std)							
	BV/TV	BS/BV	Tb.Pf	SMI	Tb.Th	Tb.N	Tb.Sp	DA
TCP/HA (60/40)	10.7 (3.8)	90.1 (18.1)	−3.72 (2.4)	1.46 (0.091)	0.098 (0.019)	1.07 (0.27)	0.37 (0.092)	0.285 (0.075)
Bio-Oss	9.4 (2.3)	95.7 (14.4)	−5.89 (3.0)	1.02 (0.11)	0.077 (0.015)	1.23 (0.30)	0.37 (0.067)	0.402 (0.072)
*P* value	0.45	0.53	0.16	<0.0001	<0.0001	0.31	0.960	0.012

**Table 3:** Micro-CT results after 8 weeks of implantation.

Group (*n* = 7)	Average (std)							
	BV/TV	BS/BV	Tb.Pf	SMI	Tb.Th	Tb.N	Tb.Sp	DA
TCP/HA (60/40)	26.0 (12.4)	38.3 (7.3)	−8.86 (6.5)	1.74 (0.80)	0.17 (0.015)	1.50 (0.73)	0.35 (0.13)	0.43 (0.074)
Bio-Oss	13.2 (2.3)	94.0 (18.0)	−5.5 (1.3)	1.27 (0.18)	0.112 (0.031)	1.23 (0.23)	0.264 (0.034)	0.415 (0.039)
*P* value	0.02	<0.0001	0.2	0.16	0.0005	0.37	0.11	0.69
